# Methodological Principles of Nasal Food Challenge

**DOI:** 10.3390/nu15173816

**Published:** 2023-08-31

**Authors:** Edyta Krzych-Fałta, Monika E. Czerwińska, Sławomir Białek, Konrad Furmańczyk, Bolesław Samoliński, Błażej Grodner, Adam Sybilski, Grażyna Nowicka, Oksana Wojas

**Affiliations:** 1 Department of Basic Nursing, Medical University of Warsaw, 02-097 Warsaw, Poland; edyta.krzych-falta@wum.edu.pl; 2 Department of Biochemistry and Pharmacogenomics, Medical University of Warsaw, 02-097 Warsaw, Poland; monika.czerwinska@wum.edu.pl (M.E.C.); blazej.grodner@wum.edu.pl (B.G.); grazyna.nowicka@wum.edu.pl (G.N.); 3 Institute of Information Technology, Warsaw University of Life Sciences, 02-776 Warsaw, Poland; konfur@wp.pl; 4 Department of Prevention of Environmental Hazards, Allergology and Immunology, Medical University of Warsaw, 02-097 Warsaw, Poland; boleslaw.samolinski@wum.edu.pl (B.S.); oksana.wojas@wum.edu.pl (O.W.); 5 2nd Department of Pediatrics, Centre of Postgraduate Medical Education, 01-813 Warsaw, Poland; adam.sybilski@cmkp.edu.pl

**Keywords:** egg allergy, nasal food challenge, optical rhinometry, VAS scale

## Abstract

Thanks to their valuable assessment possibilities (subjective complaints and changes in nasal patency during the examination), nasal provocation tests may serve as an alternative tool for oral food challenges in the future. However, this test requires successive attempts to regulate its methodology in order to develop a standardized lyophilisate form and determine the threshold dose for a positive result. The study objective was to present the methodological foundation for nasal food allergen provocation tests induced by freeze-dried powdered chicken egg whites. A control group of 25 individuals with no history of allergy to chicken eggs or any other allergy was included in the study. Optical rhinometry and visual analog scales were used to assess the response of nasal mucosa to local allergen challenges. Minor variations in nasal flows, as measured by optical rhinometry, were observed in the provocation tests. The mean optical density measurements (as measured regardless of the allergen dose used) varied from positive to negative values and vice versa, e.g., amounting to 0.018 OD (standard deviation 0.095) at 15 min and −0.011 OD (standard deviation 0.090) at 30 min. No significant differences were observed concerning the perceived nasal discomfort using the visual analog scale. Due to the absence of nasal mucosal reactivity, nasal challenge is an excellent methodological tool for implementing food allergen tests.

## 1. Introduction

A food allergy is defined as an abnormal, excessive reaction that occurs every time a subject is exposed to a specific food at a dose tolerated by healthy individuals [1]. Unlike food intolerance, food allergy is an IgE-mediated or non-IgE-mediated adverse immune response. Although immediate hypersensitivity plays the most important role in the pathogenesis of food allergy, cytotoxic reactions, and immune complex-type reactions may also be involved. The pathophysiology of food allergy results from complex interactions between the gastrointestinal mucosa, local and systemic immune responsivity, and the microbiome [1,2,3]. The first case reports on food allergies date back to the turn of the 20th century. Currently, the prevalence of food allergies in developed countries is estimated at 8% in children and ca. 5% in adults [3,4]. A worrying upward trend has been observed in the global prevalence of this pathology in recent years [1].

The nasal provocation test belongs to the group of methods in which the course of the examination is evaluated based on subjective measures for reporting complaints (e.g., visual-analog scale), as well as on objective techniques for assessing nasal patency (e.g., optical rhinometry) [5]. It is one of the few available allergological testing methods that reproduce the natural response of the mucous membrane to the local application of an allergen in controlled conditions. The response may be either IgE-mediated or eosinophilic. Early and late phases are observed in allergic reactions, resulting from a cascade of events regulated by the sympathetic and parasympathetic sensory systems as well as the receptors present within the nasal epithelium (histamine, leukotriene, prostaglandin receptors) [5,6,7,8,9]. Following a series of complex reactions, the allergen is presented (by the allergen-presenting cells, APCs) to Th2 lymphocytes, resulting in the release of IL-4 and IL-13, which in turn stimulate B lymphocytes to produce IgE and cytokines that mobilize eosinophilic cells for their specific migration within the submucosal layer as well as the mucosa. This leads to complex consequences, including the allergen being bound by sIgE and coating the mast cells within the effector tissue, contributing to the release of preformed mediators, including tryptase. Simultaneously, mast cells synthesize and release mediators, such as the platelet activation factor (PAF), LTC4, and LTD4 formed from arachidonic acid, PGD2, PGI2, and substance P. In the late phase, the stimulated cells release cytokines and chemokines into the blood, resulting in eosinophilic leukocytes and their precursor release from bone marrow. Inflammatory cell infiltrates are observed within nasal mucosa due to the inflow of eosinophils and the proliferation of precursor cells [6,7,8]. Due to the cognitive potential of nasal provocation tests, increased attention has focused on possible broadening of the indications for its implementation, including diagnostics for food allergies. Evidence is available for local response to food allergens within the nasal mucosa in nasal food allergen provocation tests [6,7,8]. Furthermore, typical symptoms—such as those observed in the early allergic reaction phase—are observed in nasal food challenge (NFC) tests with food allergens. The aim of the study was to assess the risk of nasal mucosal hypersensitivity in the nasal challenge test using incremental doses of raw chicken egg white allergen as a premise for developing and implementing NFC in the procedure of diagnosis of food allergies. The study is a research experiment and is one of the first of its kind to detail the NFC methodology, including the extraction of a substance (chicken egg) for intranasal application of an allergen. It provides, in a way, a foundation for further research in the area of standardization of NFC and expansion of its capabilities in the differential diagnosis of food allergy, as highlighted in EAACI’s consensus statement Position paper on the standardization of nasal allergen challenges. One of the indications in this document for performing nasal allergen challenge tests is further evidence diagnosing food allergy [5].

## 2. Materials and Methods

### 2.1. Ethical Statements

The study was conducted in accordance with the guidelines in the Declaration of Helsinki. Approval of the Bioethics Committee of the Medical University of Warsaw (decision no. KB 63/2022) was obtained. All participants provided signed informed consent.

### 2.2. Study Participants

The study consisted of 25 healthy adults (18 women and 7 men, residents of a large urban agglomeration), not allergic to common environmental or food allergens, as described next (Table S1). The study group demonstrated significant variability in terms of height and weight, and the majority of study participants were noted to be residents of large urban agglomerations. The selection of the group was targeted and was mainly based on a negative clinical history of allergic diseases (including, but not limited to, chicken egg allergy) and screening tests in the area of skin prick tests (a panel of 13 common allergopharma environmental allergens was used: birch, alder, hazel, grasses/corn, rye, mugwort, plantain, *Alternaria tenius*, *Cladosporium herbarum*, *Dermatophagoides pteronyssinus*, *Dermatophagoides farinae*, dog, cat, and food allergens (chicken egg, milk, soy, histamine, negative control).

Skin testing followed EAACI guidelines: Standardized allergen extracts were applied to the inner side of the forearm, preserving adequate spacing, and punctured with a standardized lancet. A positive reaction (reaction ≤ 3 mm) was assessed based on the control solution and histamine test [10,11]. Moreover, the assessment of inflammation risk in the nasal mucosa was performed using a physical examination, including endoscopy of the nasal cavity, as well as by measuring the acoustic rhinometry (MCA-1, MCA-2; Minimal Cross Sectional Area for nasal vestibule and nasal concha), spirometry (FEV_1_/FVC, FEV_1_) and nitric oxide (FENO) concentration in the air exhaled from the upper respiratory tract (Table S1). The examination was carried out at the Department of Allergology and Immunology of the University Clinical Center of the Medical University of Warsaw by qualified medical personnel: an allergologist and a nurse who both possessed the necessary knowledge, skills, and equipment for providing first aid if any life-threatening complications occur. The study group was recruited by special announcements (patient information sheet) enclosed in the documentation submitted to the Bioethics Committee of the Medical University of Warsaw.

The exclusion criteria included bacterial infection within the nasal and sinus cavities, severe comorbidities (circulatory or respiratory), severe systemic diseases (malignant tumors, autoimmune diseases), systemic immunotherapy, and pregnancy [5,9]. The study was performed with reference to the NFC protocol (Table 1) in accordance with the current standard for nasal challenge tests. After local acclimatization to room conditions (temperature 21 °C, relative humidity 43%), the examination was performed per the recommendations of the consensus on the standardization of challenge tests [5]. It includes assessment using objective techniques, including RO and the VAS scale. The measurements were carried out continuously, and the allergen in increasing doses was administered at intervals of 15 min. The added value was the allergen extracted in laboratory conditions from a solid form (hen egg) to a lyophilized form.

### 2.3. Data Collection

**Figure 1:**
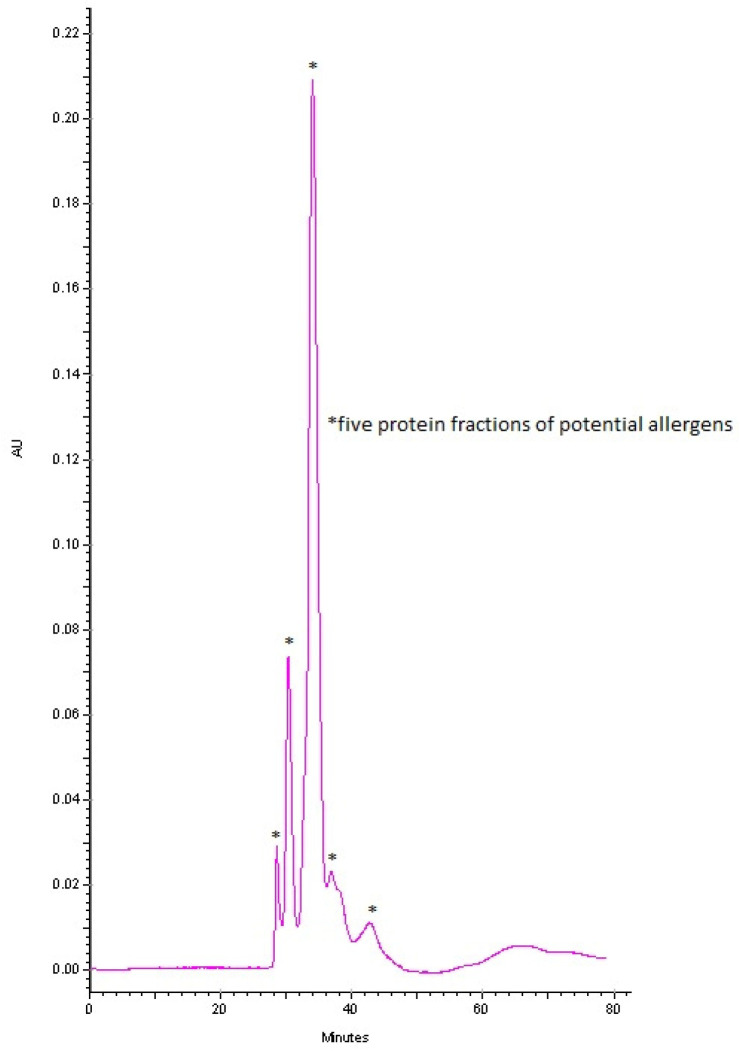
Electrophoregram of individual protein fractions within the raw egg white lyophilizate.

The NFC extract was prepared from two raw chicken eggs purchased from an organic chicken farm to ensure the absence of admixtures that could ultimately affect the test results. Whites obtained from the two eggs (total weight 50 g) were separated from the yolks and mixed with phosphate-buffered saline in a 1:4 (m/v) ratio. Aliquots of mixtures were then homogenized for at least 30 s; the homogenates were sequentially filtered through a polyethersulfone (PES) bottle top filter with a diameter of 90 mm and pore size of 0.45 µm (qpore Plastic Vacuum Filter, Bionovo, Legnica, Poland) as well as cellulose acetate (CA) filters with the diameter of 50 mm and pore size of 0.22 µm (qpore Plastic Vacuum Filter, Bionovo, Legnica, Poland) under reduced pressure (Ca-MI New Askir20, Frazione Pilastro, Italy). Following filtration, the protein was lyophilized (Christ LCG Lyo Chamber Guard, Alpha 2-4 LSC plus, Martin Christ Gefriertrocknungsanlagen GmbH, Germany) to achieve 6.6 g of freeze-dried raw chicken egg white powder. The powder was then used for the preparation of doses to be used in the challenge test. Since the weight of the stock raw egg white was 50 g, it was calculated that 1 g of raw egg white translated to 135 mg of the lyophilizate; this amount was subsequently dissolved in 1 mL of PBS. The presence of five protein fractions of potential allergens was confirmed by capillary electrophoresis (Beckman Coulter P/ACE MDQ) (Figure 1). Separation of the lyophilizate was carried out using capillary electrophoresis in a phosphate buffer environment at a concentration of 100 mM and pH = 2.5. The detection was carried out at a wavelength of 200 nm and a separation voltage of 10 kV.

The burette method determined the total protein content in 135 mg/mL of RCEWP. Thus, 135 mg of lyophilizate contained 88.4 mg of pure protein. First, the stock solution of RCEWP at a concentration of 1.52 mg/mL was prepared in PBS. The following doses of pure freeze-dried powdered chicken egg proteins were predefined within the NFC protocol: 200 µg/305.4 µg dry weight (d.w.) of RCEWP, 150/229.0, 100/152.7, 50/76.3, 25/38.2, and 12.5/19.1 µg/µg. The samples were dissolved in 200 µL of saline for intranasal application.

### 2.4. Study Design and Setting

As part of the NFC, the allergen was administered intranasally using a calibrated atomizer (maximum variation in the intranasally applied volume of ±10%, standard deviation of ±4%) in the amount of 0.02 mL bilaterally into each nostril. The assessments were made as per the standard protocol 15 min after local application using the OR technique and a 10-cm VAS scale to evaluate nasal symptoms, including itching, watery discharge, and nasal obstruction (for the total nasal symptoms: ≥55 mm extremely positive NFC, ≥23 mm moderately positive) [4].

Optical rhinometry (transmission spectroscopy: Rhinolux; Rhios GmbH, Grosserkmannsdorf, Germany) is an objective examination technique for assessing the response of nasal mucosa. The principle of the technique is an overall (bilateral) measurement of blood passing through the blood vessels within the nasal cavity (swelling of the nasal cavity is characterized by increased blood volume). This volume change indicates increased light absorbed by the mucous membrane in OR within a predefined period, using a beam of infrared radiation at a specified wavelength (600–800 µm). The device provides a continuous measurement of blood flow changes and thus provides an objective assessment. A beam of infrared radiation is emitted from the transmitter placed on the nasal bridge in a manner resembling the placement of eyeglasses. The optical curve precisely determines the onset of the response (T1) and the peak response to the locally applied allergen (T2). The optical density (OD) parameter is important for defining strongly positive, moderately positive, or negative test results. In the case of a positive response to the applied allergen, an increase in the absorption of infrared radiation—and thus an upward trend of the optic curve—is observed. On the other hand, negative values are observed in tests involving, e.g., the use of xylometazoline; alternating negative and positive values are observed within a particular time frame in the nasal cycle. Positive results of allergen challenge tests were defined based on the OD, as measured by OR increasing by 0.52 [12,13] and based on VAS scores of at least 55 mm [5,9]. The total duration of the study was 120 min. The study was performed by qualified personnel in hospital conditions. The study was conducted and funded under the National Science Center Miniature-5 grant (2021/05/X/NZ5/01/099).

### 2.5. Statistical Analyses

**Figure 2:**
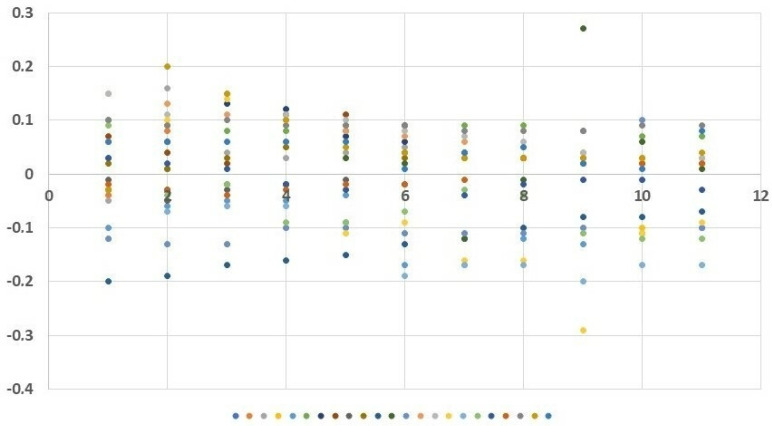
Distribution of ΔE within the time range of 5–55 s (each bar represents the distribution of ΔE values obtained from the study subjects at a specific time point; the measurement of ΔE for each patient is marked with a different color).

**Figure 3:**
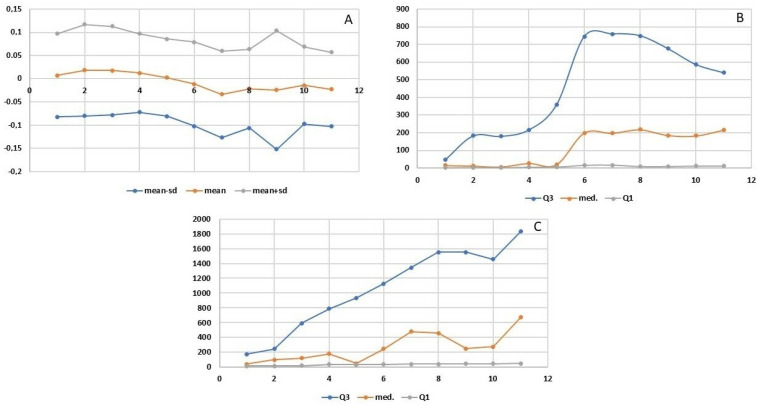
Optical rhinometry in nasal food challenge: (**A**) Optical density (mean, mean minus standard deviation (sd), mean plus standard deviation (sd) of ΔE for each specific time point within the range of 5–55 s); (**B**) Flow variability onset (T1) [1st quartile (Q1), median, 3rd quartile (Q3) for T1 values]; (**C**) Temporal peak of flow variability (T2) [1st quartile (Q1), median, 3rd quartile (Q3) for T2 values].

Statistical analyses included basic descriptive statistics (mean, standard deviation (SD)] as well as positional statistics (quartiles). An analysis of empirical distributions of the OD differences (ΔE) and the T1 and T2 parameters is also presented as variability charts and box plots (Figure 2 and Figure 3A,B). We used the Welch Two Sample *t*-test for comparisons. Statistical significance was determined with a *p*-value < 0.05.

## 3. Results

### 3.1. Variability of Nasal Flow at Individual Time Points in Relation to an Increasing Dose of Allergen Administered

**Figure 4:**
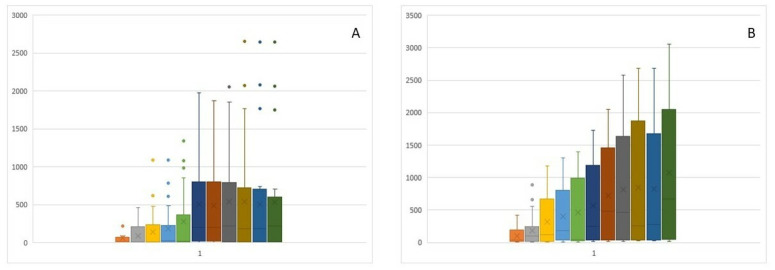
Distribution of empirical study parameters: T1 and T2: (**A**) Box plots for individual T1 measurements; and (**B**) Box plots for individual T2 measurements (the colors represent the particular measurement from 5 to 55 min).

The increasing doses of the allergen did not cause any statistically significant subjective ailments as measured by the VAS scale. Nasal itching was the only symptom observed at 0.95 mm in two subjects following the administered dose of 50 µg. Slight variability of nasal flows as measured employing OR was observed during the early allergic reaction phase, following the local application of the saline solution and the increasing doses of the allergen (12.5, 25, 50, and 100 µg). The mean values of the OD (measured regardless of the allergen dose used) were both positive and negative: 0.007 OD (SD 0.089) at 5 min, 0.019 OD (SD 0.098) at 10 min, 0.018 OD (SD 0.095) at 15 min, 0.012 OD (SD 0.084) at 20 min, 0.003 OD (SD 0.083) at 25 min, −0.011 OD (SD 0.090) at 30 min, −0.033 OD (SD 0.093) at 35 min, −0.021 OD (SD 0.085) at 40 min, −0.024 OD (SD 0.128) at 45 min, −0.014 OD (SD 0.083) at 50 min, and −0.023 OD (SD 0.080) at 55 min (Figure 3 and Figure 4A). Similarly, no statistically significant differences were observed at predefined time points corresponding to the assessment of mucosal response to the allergen used (at 15, 30, 45, and 50 min into the study). The slight variability observed in the assessment was identical to that observed in the nasal cycle.

### 3.2. Mean Optical Density, Onset, and Greatest Change in Flow Measured by Optical Rhinometry

The onset of flow variability was observed at 8.48–8.97 min into the study, whereas the peak change was observed at 13.73–17.88 min (Figure 3B,C). Due to the low variability of nasal flows during the early allergic reaction phase, no significant differences were recorded at individual study time points of 15, 30, 45, and 60 min after application. The T1 and T2 parameters (Figure 3B,C) are characterized by high variability of the third quartile and median values at individual measurement points. The wide distribution of these value results translates to the absence of significant differences being observed in these parameters over time. This effect is evident in the box plot charts in Figure 4A,B.

## 4. Discussion

### 4.1. Benefits of Nasal Provocation Test in Diagnosing Food Allergies

Among various indications for NFC, the following are worth noticing: diagnosis of persistent, chronic, occupational, and local allergic rhinitis, identifying allergy to airborne allergens, determining the cause-and-effect relationship between the allergen and symptoms, especially in cases when interpretation of skin test results and sIgE concentration are difficult, determining the indications for immunotherapy, identifying allergens directly responsible for the symptoms and designing effective vaccine based on these findings, as well as monitoring the effectiveness of desensitization and pharmacotherapy, performing differential diagnosis of eye symptoms and finally, using the obtained information for scientific purposes (studying the pathophysiology of allergic reactions and examining the influence of various factors on the course of this reaction) [5,9]. An additional indication stated by the consensus of experts involving the implementation of nasal provocation tests in diagnosing food allergy appears to be particularly important for future clinical practice [5]. Currently, little data is available in the literature on the use of nasal allergen provocation tests in the diagnostics of food allergies. In this study, the present authors presented a detailed protocol of the NFC test as a potentially useful and safe tool in diagnosing food allergy. The diagnosis of a food allergy is complex due to the rich symptomatology and the multitude of symptom-triggering factors. The first documented report on the diagnosis of food allergy is from 1912, when scratch tests were performed and probably first reported by an American pediatrician, Oscar Menderson Schloss [14]. In the following years, the diagnostic methods used in this disease unit continued to develop and improve. Currently, the standards for managing suspected food allergies include an interview, physical examination, skin tests, laboratory analyses, and component diagnostics. However, a double-blind, placebo-controlled food challenge remains the gold standard [1,2,3,4]. The main indication for the challenge test is to confirm the causal relationship between the consumption of a particular food and the development of hypersensitivity symptoms. As a result, the causative role of the suspected food product in the development of the disease can be confirmed. Another important indication for the test is documentation of the development of tolerance to food products that had previously triggered hypersensitivity reactions, including the development of tolerance following specific immune therapy [1,15]. The main advantage of the challenge test is the fact that it reproduces the natural route of exposure to the allergen. Nonetheless, the oral challenge test is fraught with the possibility of burdensome, severe, and potentially life-threatening symptoms and should be carried out in hospital conditions, thus substantially increasing the procedure costs. Another important limitation is the necessity to precisely blind the examined foods [16].

During oral challenge tests, patients were found to frequently present with nasal symptoms—such as itching, sneezing, watery discharge, and nasal obstruction—in addition to symptoms of the digestive tract and skin. These observations have laid the foundation for research on the use of nasal challenge tests in the diagnosis of food allergies. Nasal allergen provocation tests are widely used in differential diagnostics of rhinitis. This is because such tests facilitate confirmation of the causal role and identification of factors responsible for IgE-dependent hypersensitivity reactions in allergic rhinitis, as well as confirmation of pharmacotherapy and specific immune therapy as used in treating allergic rhinitis. Nasal allergen provocation tests are relatively safe and can be performed in outpatient settings. The immediate reaction symptoms usually resolve within several minutes. Assessment of the challenge tests is based on clinical evaluation and objective measurement techniques, including rhinomanometry, acoustic rhinometry, peak nasal inspiratory flow (PNIF), and OR [5,17]. These considerations have instigated the search for safer, more accessible, and possibly outpatient-based diagnostic methods (compared with oral food challenge) that would facilitate reproduction of the natural route of allergen exposure. Kvenshagen and Jacobsen assessed the possibility of diagnosing food allergies using techniques other than the oral provocation test, a potentially dangerous, expensive, and time-consuming tool. According to the assumed premise, the new diagnostic tool should be cheaper, safer, and easier to perform than the oral provocation test while at the same time being painless and reliable. The authors carried out a relevant query within full-text databases (Embase, PubMed, and Cochrane) to obtain as few as seven publications on the use of mucosal allergen challenges (to nasal, conjunctival, labial, and gastrointestinal mucosa as assessed by endoscopic means) in the diagnostics of food allergies to be included in the analysis. Due to the availability of mucous membranes and the possibility of using small doses of allergen, they found these methods promising but undoubtedly requiring validation and standardization to be used as alternatives to the oral provocative tests [18]. A peculiar basis for using intranasal provocation tests in food allergies was laid by Amlot et al. in 1985. The authors presented the results of a study involving the use of nasal, labial, and gastric provocation in 39 patients with oral allergy to milk and hen’s eggs as diagnosed from the clinical history of positive skin prick test results. The results of the nasal provocation tests were assessed based on PNIF measurements and the number of sneezes. No oral food challenge test was performed. Based on the obtained results, the nasal allergen provocation test was considered to be the most sensitive modality [19]. In the following years, only three other papers were published on the subject. Studies on nasal challenge tests with chicken egg and peanut allergens were published in 1993 by Seppey et al. and later in 2007 and 2012 by Clark et al. Facial thermography was used by the authors to assess the test results; the examination was considered rapid, safe, and objective. However, no further studies were conducted [20,21,22]. A breakthrough was made in 2021 when Gelis et al. presented the results of an innovative study assessing the usefulness of the nasal allergen provocation test in the diagnostics of shellfish allergies and in the differentiation of patients with allergy and non-allergic hypersensitivity as an alternative to the oral food challenge test. Included in the study were 45 people with a shrimp allergy confirmed employing a skin prick test, nasal allergen provocation test, and history of anaphylaxis or intolerance of shrimp in medical history. The control group consisted of 10 healthy individuals. Boiled shrimp lyophilizate was used for the nasal test, and the results were assessed based on acoustic rhinometry and a visual analog scale. The results confirmed the usefulness of the nasal allergen provocation test in diagnosing shrimp allergies [23].

An allergy to chicken egg proteins is one of the most common food allergies encountered in an allergologist’s practice. The incidence of chicken egg protein allergy in infants and young children ranges from 0.5% to 2% [24]. As revealed by the results of a multicenter cohort Europrevall study carried out from 2005–2009, the incidence of chicken egg allergies in children aged up to 2 years was 1% [23,25]. In adults, food allergy to chicken eggs is much less common and accounts for 0.1%. It is believed that persistent allergy is the main cause of presentation in adults, whereas primary hypersensitivity to egg proteins is rare. The allergy to chicken eggs is particularly challenging to patients and their families due to the necessity of abstaining from numerous food products and fear of accidental use; it is also an unfavorable prognostic factor of future inhalant allergens as well as the development of asthma and allergic rhinitis [26,27]. For this reason, an accurate diagnostic process is important, as is the safety of diagnostic procedures. Herein, the present authors describe the possibility of using freeze-dried chicken egg white and yolk powder in NFC and reveal the lack of irritating effects of this form (raw chicken egg) on the nasal mucosa.

### 4.2. Limitation of Study

One limitation of the study is the relatively small number of scientific reports conducted on a representative group of subjects, which would allow for including the NFC method in diagnostic guidelines for food allergies. Another issue is the lack of ready-made tools enabling scientists to prepare lyophilisate in a form that would meet the criteria for standardized nasal preparations. Those used in skin tests often contain glycerin, which is not indifferent to nasal mucosa and may potentially increase the risk of false-positive results. Therefore, using this form of allergens in NFC is impossible for methodological reasons. On the other hand, using ready-made food preparations, e.g., milk, may increase the risk of non-specific immune responses resulting from industrial technologies used in food processing [28]. The method of obtaining allergen via filtration in laboratory conditions implemented by our research team creates new opportunities for further research in this field.

## 5. Conclusions

The freeze-dried chicken egg white powder used in NFC did not increase the risk of nasal mucosal reactivity in the control group. The next research stage will be based on the NFC model developed herein, and will involve a group of subjects with allergies to chicken egg protein allergens.

## Figures and Tables

**Table 1:** The Nasal Food Challenge Testing Protocol.

	Female	Male	Urban	Rural	General
Age Mean (years),	33.5	34.4	35.2 ***	26.3 ***	33.8
Age SD	8.5	5.9	7.5	4	7.7
Height Mean (cm),	168.7 *	181.1 *	172.2	171.8	172.2
Height SD	3.9	4.1	7.0	7.4	6.9
Weight Mean (kg),	63.1 **	75.1 **	66.8	64.5	66.4
Weight SD	4.6	6.4	7.6	7.3	7.5
BMI Mean,	22.2	22.9	22.5	21.8	22.4
BMI SD	1.5	1.1	1.4	1.2	1.4
Assessment of nasal patency					
Acoustic rhinometry	Female	Male	Urban	Rural	General
MCA-1R Mean	0.351	0.347	0.355	0.323	0.350
MCA-1R SD	0.058	0.081	0.065	0.053	0.064
MCA-1L Mean	0.343	0.299	0.337	0.295	0.330
MCA-1L SD	0.063	0.062	0.068	0.030	0.065
MCA-2R Mean	1.711	1.621	1.703	1.595	1.686
MCA-2R SD	0.423	0.423	0.422	0.357	0.407
MCA-2L Mean	1.676	1.630	1.704 ****	1.448 ****	1.663
MCA-2L SD	0.401	0.361	0.403	0.137	0.383
FENO Mean (ppb),	393.722	524.714	435.191	405.250	430.4
FENO SD	140.938	167.927	170.825	47.352	157.237
Spirometry					93.600
FEV1/FVC Mean (%),	93.389	94.143	93.476	94.250	5.346
FEV1/FVC SD	5.414	5.551	5.662	3.775	
					92.240
FEV1 Mean (L),	92.111	92.571	91.905	94.000	7.293
FEV1 SD	7.529	7.208	7.334	7.874	

* *p* = 2.775e, ** *p* = 0.001598, *** *p* = 0.00921, **** *p* = 0.03612 (the Welch Two Sample *t*-test). SD-the standard deviation.

## Data Availability

Data presented in this study are available on request from the corresponding author.

## Supplementary Materials

The following supporting information can be downloaded at: https://www.mdpi.com/article/10.3390/nu15173816/s1, Table S1: The Nasal Food Challenge Testing Protocol.
