# Supplementary material for: Methodological Principles of Nasal Food Challenge
Source: Nutrients. 2023 Aug 31;15(17):3816. doi: 10.3390/nu15173816 (PMC10489609; doi:10.3390/nu15173816)
Supplement: Supplementary file 1 [file nutrients-15-03816-s001.zip › nutrients-2562730-supplementary.pdf]

**The Nasal Food Challenge Protocol**<br>**..**	**Test date**
Name (first, last)	Gender M ☐<br>F ☐<br><br>Age ....

***Exclusion criteria**

History of anaphylactic shock; ☐	Nose deformations; ☐	Atrophic rhinitis; ☐	History of vaccination within the last week; ☐
Surgeries within the last; 8 weeks; ☐	Upper respiratory tract infections; ☐	Exacerbation of an allergic disease; ☐	Uncontrolled asthma; ☐
Lower respiratory tract diseases involving severe airway restriction or obstruction; ☐	Contraindications to adrenaline, pharmacotherapy including β-adrenolytics and ACEIs; ☐	Lack of access to equipment and medications necessary for the treatment of anaphylactic shock; ☐	Significant natural exposure to allergen resulting in a risk of anaphylaxis; ☐
Systemic immunotherapy; ☐	Severe heart disease; ☐	Severe systemic diseases; ☐	Pregnancy ☐

control solution challenge

**Subjective assessment (VAS)**	nasal itching	0 ______________________________	10
	number of sneezes	0 ______________________________	10
	discharge	0 ______________________________	10
	obstruction	0 ______________________________	10
**Objective assessment (OR)**	RMM	PA/cm^3/s.	
	PNIF	L/min.	
	RA	CSA-1 (cm^2)<br>CSA-2 (cm^2)	
	**RO	ΔE	

Notes

....

....

*allergen challenge (certain doses of allergens: 12.5, 25, 50, and 100 µg)			
**Subjective assessment (VAS)**	nasal itching 0 ———————————— 10<br>number of sneezes 0 ———————————— 10<br>discharge 0 ———————————— 10<br>obstruction 0 ———————————— 10		
**Objective assessment (OR)**	RMM	PA/cm^3/s.	
	PNIF	L/min.	
	RA	CSA-1 (cm^2)<br>CSA-2 (cm^2)	
	**RO	ΔE	

Notes

....

....

***positive challenge test**			***moderately positive challenge test**		***negative challenge test**	
VAS/≥55 mm	Objective technique		VAS/≥23 mm		VAS/no discomfort	
	RMM/≥40% flow reduction at 150 Pa		RMM/≥20% flow reduction at 150 Pa		RMM/no flow reduction	
	PNIF/≥40% flow reduction		PNIF/≥20% flow reduction		PNIF/no flow reduction	
	RA/≥40% reduction in CSA-2		RA/≥20% reduction in CSA-2		RA/no reduction in CSA-2	

	**RO/ΔE: increase by 40%		**RO/ΔE: increase by 20%		RO/ΔE: no increase	

* EAACI Position paper on the standardization of nasal allergen challenges [5], ** Wüstenberg et al./0.52 OD [12]

Table S1. The Nasal Food Challenge Testing Protocol.
